# Data Analysis Methods for Synthetic Polymer Mass Spectrometry: Autocorrelation

**DOI:** 10.6028/jres.107.005

**Published:** 2002-02-01

**Authors:** William E. Wallace, Charles M. Guttman

**Affiliations:** National Institute of Standards and Technology, Gaithersburg, MD 20899-8541

**Keywords:** autocorrelation, correlation function, data analysis methods, informatics, mass spectrometry, polymer, time series

## Abstract

Autocorrelation is shown to be useful in describing the periodic patterns found in high- resolution mass spectra of synthetic polymers. Examples of this usefulness are described for a simple linear homopolymer to demonstrate the method fundamentals, a condensation polymer to demonstrate its utility in understanding complex spectra with multiple repeating patterns on different mass scales, and a condensation copolymer to demonstrate how it can elegantly and efficiently reveal unexpected phenomena. It is shown that using autocorrelation to determine where the signal devolves into noise can be useful in determining molecular mass distributions of synthetic polymers, a primary focus of the NIST synthetic polymer mass spectrometry effort. The appendices describe some of the effects of transformation from time to mass space when time-of-flight mass separation is used, as well as the effects of non-trivial baselines on the autocorrelation function.

## 1. Introduction

The advent of rapid, high-resolution, broadmass-range mass spectrometry has revolutionized synthetic polymer single-chain characterization [1]. Along with this new measurement technology has come a flood of high-quality mass spectral data of an exceedingly complex nature. It is not unusual for synthetic polymer mass spectra to contain hundreds of separate peaks even when excluding those simply derived from naturally-occurring isotope distributions. Automated data analysis methods are needed in order to make full and timely use of the data.

Time series analysis, which first came to fore with the publication of Norbert Wiener’s seminal text *Extrapolation, Interpolation, and Smoothing of Stationary Time Series with Engineering Applications* [2] in 1949, has proved invaluable in many fields of data analysis. Weiner’s text represents the first complete exposition of the study of operations on time series, including autocorrelation and cross-correlation. In the intervening years these correlation methods have been applied to many types of mass spectral data for many purposes [3–5]. Owens has reviewed the use of correlation functions in mass spectroscopy, in particular, the use of autocorrelation and cross-correlation as applied to ion fragments in order to identify small organic molecules in standard libraries [6]. Hercules and coworkers have used autocorrelation of isotope distributions as a method to optimize automated data collection [7]. Here we discuss the application to synthetic polymer mass spectra for the purpose of efficiently extracting information from complex data.

First we define the mass autocorrelation and show how to treat the data properly for its use. Then we present autocorrelation for a spectrum of a simple polyethylene oxide homopolymer to establish the fundamentals. Following that we present data on two more complicated structures, specifically two silsesquioxanes produced by condensation polymerization [8] in which the mass spectra can be related directly to the polymer architecture. Finally, we apply autocorrelation to the issue of quantitation in polymer mass spectrometry using the example of polybutadiene.

## 2. The Mass Autocorrelation Function

We define the mass autocorrelation function as

$$G(L)=\Sigma_{i}S(m_{i})S(m_{i+L})/\Sigma_{i}S(m_{i})S(m_{i})$$ (1)

where *S*(*m_i_*) is signal at mass *m_i_* taken on equal intervals of mass, δ*m*. Equal intervals of mass are used because most correlation algorithms, and the closely related field of fast Fourier transforms (FFT), require the signal to be evenly spaced points on the scale of interest.

Time-of-flight (TOF) mass separation [9] is the technique most often applied to synthetic polymers due to their high molecular masses, typically in excess of 1000 u and often much greater (into the 100 000 u range and beyond). No other mass separation technique can reach such high masses. The TOF signal, *s*(*t_i_*), is collected on equal intervals of time. The transformation from this time-base signal *s*(*t_i_*) to a mass-base signal *S*(*m_i_*) involves both an interpolation and a change of the signal itself by a Jacobean transform. The mathematics to affect this transformation is discussed in Appendix A.

## 3. Example 1: A Simple Linear Homopolymer

**Fig. 1:**
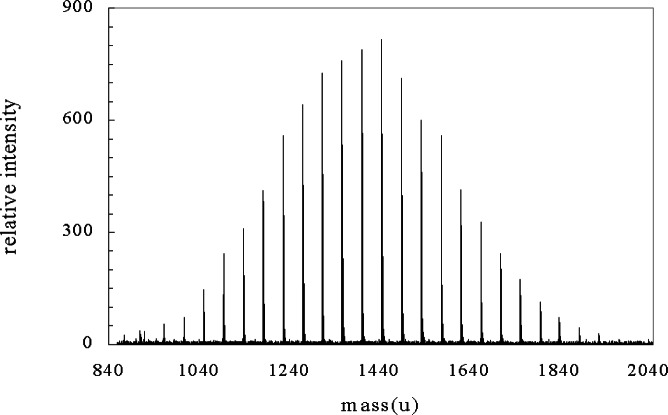
Matrix-assisted laser desorption/ionization time-of-flight mass spectrum from a polyethylene oxide of a molecular mass centered around 1440 u.

**Fig. 2:**
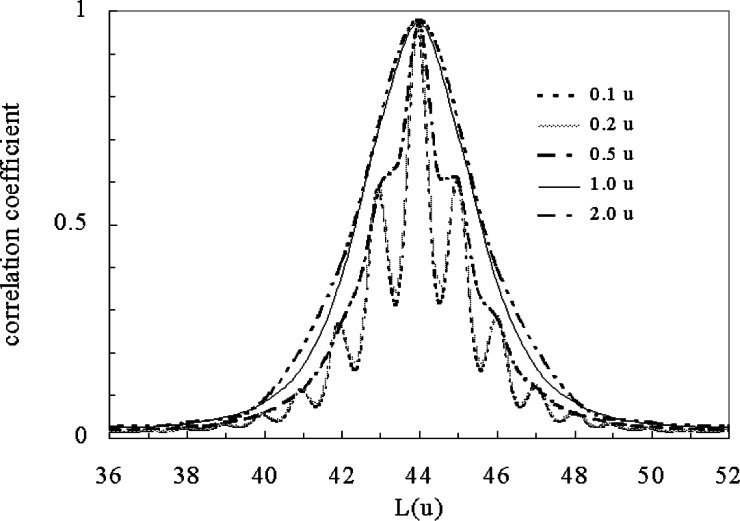
Mass autocorrelation of the data in Fig. 1. The effect of various coarse graining parameters on the representation of the data is seen. Notice as δ*m* increases above 0.5 u the isotope resolution is lost.

The most obvious use of mass autocorrelation function is to get an accurate representation of the repeat unit of the polymer. This can be difficult in a spectrum with noise where identification of peak position will inevitably lack precision and lead to inaccuracies in calculating the repeat unit mass. Figure 1 shows the mass spectrum for a low-molecular-mass polyethylene oxide (repeat unit: [–CH_2_–CH_2_–O–]); while Fig. 2 is its autocorrelation function with different values of δ*m*. Data were obtained by matrix-assisted laser desorption/ionization (MALDI) TOF mass spectrometry [10, 11]. Before autocorrelation a baseline was pulled off the data in time space and the data was subsequently transformed from time space to mass space by the partial integration method described in Appendix A. The autocorrelation clearly shows the 44.03 u repeat unit of polyethylene oxide with a precision difficult to match by simply picking adjacent peaks and calculating a mass difference.

Now consider the effect of varying the δ*m* for partial integration or interpolation. The spectrum and its autocorrelation function with δ*m* chosen to be from 0.1 u to 2.0 u are also shown in Fig. 2. It is clear we get a varying representation of the repeat unit and its isotope effect depending on the choice of δ*m*. By increasing δ*m*, that is, by integrating over a wider window of the data for each point, we obtain less sensitivity to the isotopes, that is, a greater smoothing effect on the data but less accuracy in peak position.

## 4. Example 2: A Complex Homopolymer

Polysilsesquioxanes are three-dimensional polymers with a tri-functional repeat unit of the form [RSiO_3/2_] where each silicon atom is coordinated with three oxygen atoms. They are most often produced by a low temperature sol-gel hydrolysis-condensation reaction from silicon alkoxides [12]. One important unknown in the processing of silsesquioxanes is the “degree-of-condensation” as a function of molecular mass. That is, how many of the silicon atoms are three-fold coordinated with bridging oxygen atoms and how many have terminal silanol (≡SiOH) groups?

**Fig. 3:**
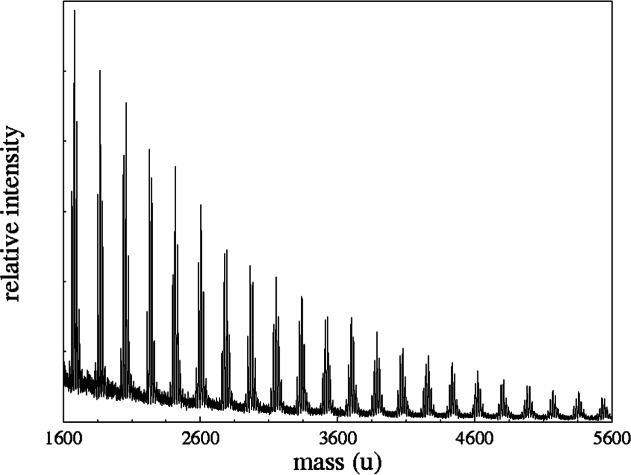
The full mass spectrum of the methacrylpropyl silsesquioxane showing the characteristic shape of a condensation polymer. Estimated standard uncertainty (Type A) of the peak position from calibration and repeatability studies is 0.2 u, and the estimated standard uncertainty in overall signal intensity from repeatability studies is 15 %.

The mass spectrum of methacrylpropyl silsesquioxane (R = (CH_2_)_3_–O–CO–CCH_2_–CH_3_) is seen in Fig. 3 [13, 14]. Each major cluster of peaks corresponds to a single oligomer with a given number of repeat units *n*. Since the monomer contains one silicon atom the value of *n* also corresponds to the number of silicon atoms in that oligomer. For this material this average mass of the basic repeat unit is 188.25 u. (The average is taken over all isotopes of each element present using their natural abundances.) This is the value of the mass difference between groups of peaks seen in Fig. 3. Knowing that ionization occurs via the attachment of adventitious Na^+^, and by including the mass of the two O_1/2_ H end groups, an exact identification of each oligomer present in the sample can be made.

**Fig. 4:**
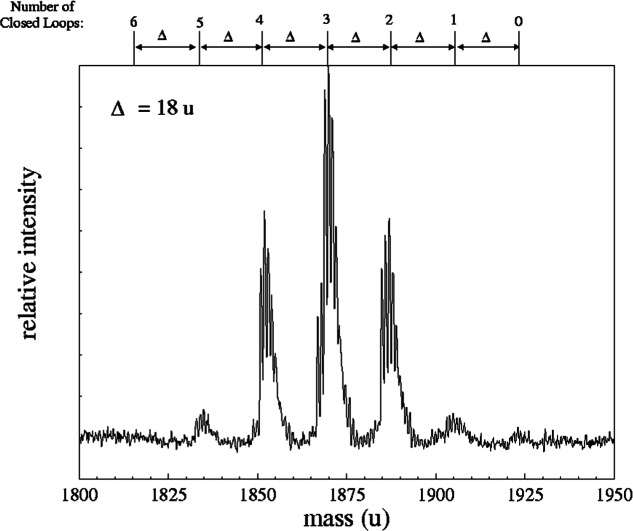
Detail around a single oligomer of the methacrylpropyl silsesquioxane from Fig. 3 for *n*=10. Across the top of the figure is given the number of closed loops *t* indicated by the loss of water (18u). The maximum value for *t* was 3 with the lowest *t* being 0 and the highest being 6.

Figure 4 shows the detail of a single low-mass oligomer from Fig. 3. The maximum possible mass of an oligomer with *n* repeat units occurs when every silicon atom has one silanol group in addition to one R-group and two bridging oxygen atoms. Two bridging oxygen atoms are the minimum number necessary for the formation of a polymer, that is, conceptually polymerization requires difunctionality at a minimum. Thus, the repeat unit in this case can be given as [RSi(O_1/2_)_2_OH]. For an oligomer with *n* repeat units the mass of the heaviest oligomer is *n* times the mass of this “difunctional” oligomer (plus the mass of the Na^+^ ion and the end groups). This heaviest oligomer is the linear or branched structure. However, the highest intensity peak generally does not appear at the maximum possible mass. Instead, lower mass peaks are more intense. These peaks correspond to the loss of water as pairs of Si-OH groups react. This in turn immediately indicates that intramolecular reactions are occurring during polymerization. If *intermolecular* reactions were occurring the value of *n* would change and a new, higher mass, oligomer would be formed. In Fig. 4, *n* = 10 and the number of closed loops *t* is given across the top of the figure. The value of *t* ranges from 0 to 6 with 3 being the most likely value. Note that each peak is separated by 18 u indicating the loss of water.

For the condensation polymer derived from the silsesquioxane monomer considered here, the mass *m* of the *linear* oligomer having *n* repeat groups is given in units of u by the equation:

m=(188.25)+p+18 (2)

where *n* is the number of repeat groups whose mass is 188.25 u, *p* is the mass of the cation (either 23 u for sodium, or 39 u for potassium), and 18 u is for the two O_1/2_H end groups. It is easy to show that either a strictly linear or a branched-linear polymer, which does not have one of the branches forming a closed loop with the oligomer itself, follows the above formula for mass. This formula would explain a single peak for each oligomer but cannot explain the major clusters that were observed and ascribed to intramolecular ring formation.

This suggests a modified version of Eq. (2) that includes intramolecular closed loop formation:

m=(188.25n)+p−(18t)+18 (3)

where again *n* is the number of repeat units, *p* is the mass of the cation, *t* is equal to the number of closed loops in the molecule (i.e., the number of lost water molecules), and 18 u in the last term is for the added end groups.

**Fig. 5:**
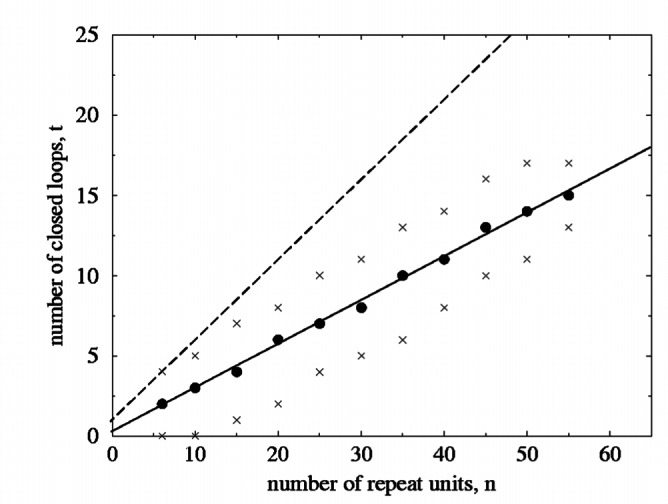
plot of the number of intramolecular closed loops, *t*, versus the number of repeat units in a given oligomer, *n*. The solid circles represent the maximum intensity peak for each oligomer, and points marked with an *x* give the maximum and minimum number of observed loops. The solid line is a linear regression fit to the solid circles, while the dashed line is the expected value *t* = 1/2*n* + 1 (for *n* even) for the fully-condensed polyhedral structure. The sample showed an intermediate behavior between a branched linear structure and a fully-condensed structure.

Applying these concepts to the full mass spectrum, Fig. 5 gives the number of closed loops *t* per oligomer with *n* repeat units, that is *t* vs *n*. The solid circles give the number of closed loops for the most intense minor cluster of each major cluster. (Recall that a major cluster corresponds to an oligomer with *n* repeat units.) The points marked with an *x* are for the least intense peaks observed in each major cluster, that is, the weakest peaks found before the baseline noise overtakes the signal. The regression fit of the solid circles given by the solid line in Fig. 5 has a slope of 0.273 with a standard uncertainty of 0.006, an intercept of 0.226 with a standard uncertainty of 0.192, and a correlation coefficient of 0.998. (The “standard uncertainty” is the estimated standard deviation of the fitted parameter.) The first observation is that the ratio of *t/n* remains roughly constant for all *n* with a value of about 1/4. This suggests that the molecule is no more or less likely to interact with itself based solely on its size. Stated another way, the molecule may be fractal-like with its closed-ring topology independent of molecular size [15]. A fully-condensed polyhedral structure with an even number of repeat units will follow the equation *t* = 1/2*n* + 1, while for an odd number of repeat units the governing equation is *t* = 1/2(*n* – 1) + 1. This is shown as a dashed line in Fig. 5 on the other hand, a branched linear chain with no closed loops will have *t* = 0 (by definition), and thus *t/n* = 0 which is merely the abscissa of the graph. Therefore, in general it appears as if the specific silsesquioxane studied has on the average an assortment of closed loops and linear branches in each molecule. No fully-condensed polyhedra were observed except at very low mass (*n*<10) because the experimentally-observed *t/n* ratio was on the order of 1/2 well below the fully-condensed-polyhedron value of (for large *n*). The analysis of this data requires analysis of each peak and identifying it with each species. This can be very laborious if one wishes to screen a large number of compounds.

**Fig. 6:**
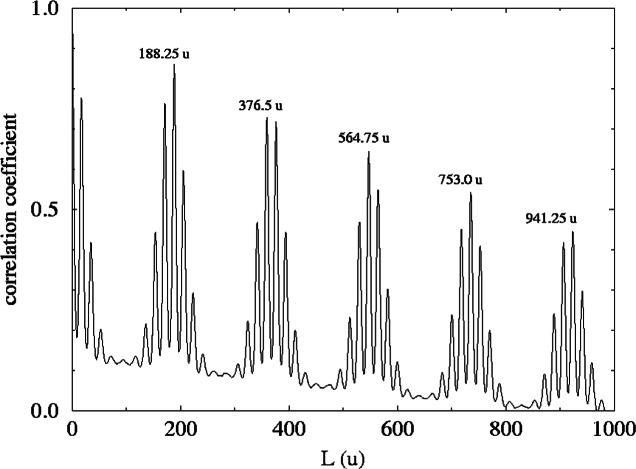
The full autocorrelation function with a lag, *L*, from (1 to1000) u for the mass spectrum shown in Fig. 3. The autocorrelation coefficient is plotted versus *L* in units of u.

**Fig. 7:**
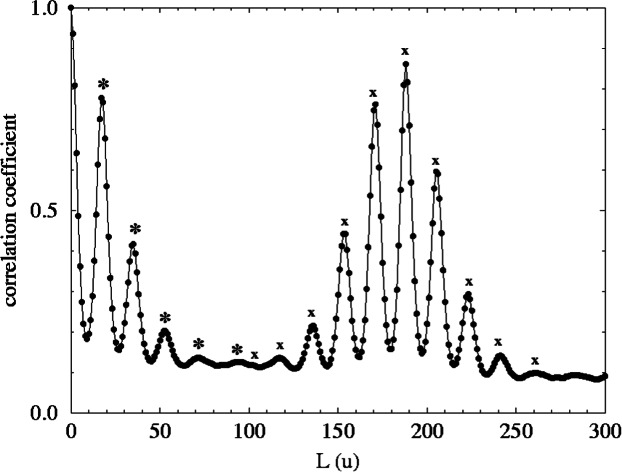
Low-mass-region detail of autocorrelation function shown in Fig. 6. The stars show the 5 peaks shifted by 18 u found in each major cluster. The positions marked with an *x* are the 10 peaks found by correlations between major clusters 188.25 u apart.

The mass autocorrelation function was applied to the data in Fig. 3 with the lag, *L*, in the range from (0 to 1000) u and with δ*m* = 1 u and is shown in Fig. 6. It largely replicates the original mass spectrum without much of the baseline noise. In this way it can be roughly thought of as a kind of “averaging.” The peaks at 188.25 u are for correlations of Δ*n* = 1, those at 376.5 u are for Δ*n* = 2, etc. Figure 7 is the low mass region of the autocorrelation function expanded. There are a series of five low mass peaks, marked with stars in the figure, starting at 18 u and each 18 u apart. This indicates that the number of closed loops per oligomer should be about five, that is, there should be five peaks in each major cluster. Recall that this was shown in Fig. 4 where the difference for each oligomer between the maximum and minimum number of closed loops observed, *t*, is about five. Likewise, in Fig. 7 the number of peaks in the autocorrelation function around mass 188.25 u should be about 10, marked with the symbol x in the figure, that is, correlations of the five peaks of two adjacent major clusters. Lastly, since the spacing used in this autocorrelation function is 1 u, the isotopic resolution that should be apparent at 1 u is not seen, instead autocorrelation within the minor peaks is simply smeared out.

**Fig. 8:**
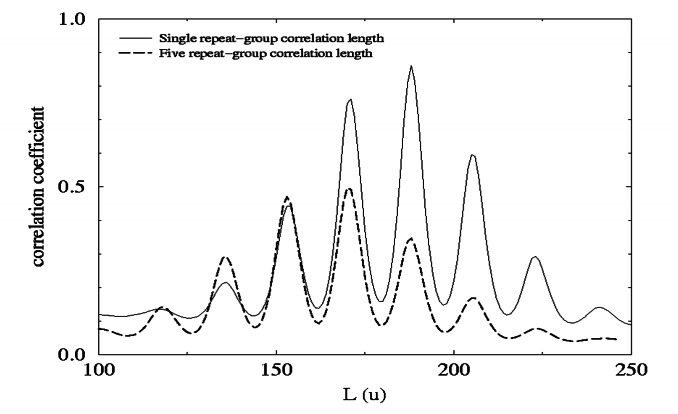
Shift of the five-repeat-unit correlation function (dashed line) onto the single-repeat-unit correlation function (solid line) showing the 18 u offset between the maximum peak for each group.

Figure 8 shows the autocorrelation function centered at 941.25 u (corresponding to correlations over five repeat unit masses, i.e., 5×188.25 u = 941.25 u) superimposed over the autocorrelation function at 188.25 u. This was done simply by subtracting 753.0 u = 4 × 188.25 u from the autocorrelation function centered at 941.25 u. Notice that the maximum peak for 941.25 u group is 18 u to the left of the maximum peak for the 188.25 u group. This indicates that as four repeat units are added to an oligomer (*n* = *n* + 4) one added closed loop is formed per molecule on average (*t* = *t* + 1). Once again this can be seen from the slope of the line in Fig. 5: for each step of *n* equal to four, *t* is increased by about one. (Strictly, since experimentally *t/n* = 0.273, an increase in *n* of 4 should yield an increase in *t* of 1.1. This is hinted at in the peak heights of Fig. 8). This provides another view that the molecule is self-affine in that adding additional repeat groups changes proportionately the number of closed loops. In contrast, a strictly linear polymer undergoing a random walk crosses itself in proportion to the square root of the number of repeat units, i.e., $t\approx \sqrt{n}$. This behavior is clearly not seen in this material. Each of these trends is revealed rapidly by mass autocorrelation and would not be as readily apparent in a peak-to-peak indexing of the data.

## 5. Example 3: A Copolymer

MALDI-TOF mass spectrometry was performed on a low molecular mass fraction of a copolymer of methyl silsesquioxane (repeat unit: [CH_3_SiO_3/2_]) and dimethysiloxane (repeat unit: [(CH_3_)_2_SiO]) monomers (Dow-Corning Metflex™)^1^. This fraction had a nominal mass of 3400 u by size-exclusion chromatography.

**Fig. 9:**
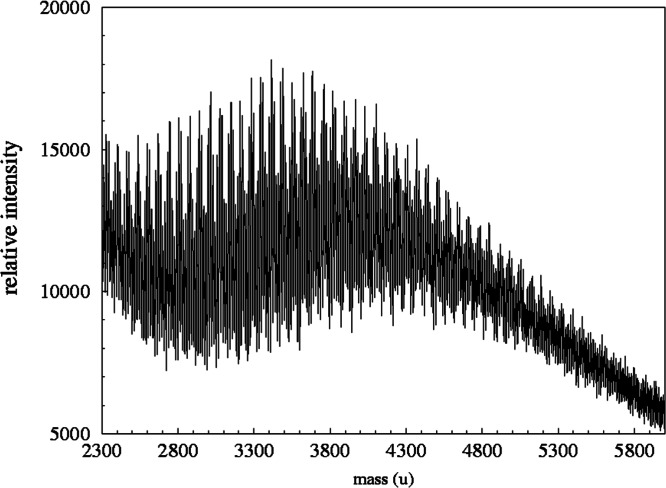
The full mass spectrum of the methylsilsesquioxane/dimethylsiloxane copolymer showing the characteristic shape of a condensation polymer. Estimated standard uncertainty (Type A) of the peak position from calibration and repeatability studies is 0.2 u, and the estimated standard uncertainty in overall signal intensity from repeatability studies is 15 %.

**Fig. 10:**
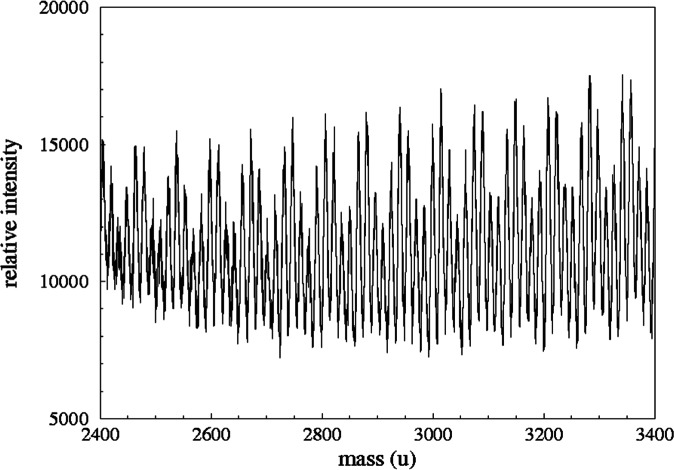
Detail of the mass spectrum shown in Fig. 9 showing the complexity of the signal.

Figure 9 shows the full spectrum of sample while Fig. 10 shows a detailed region of this spectrum highlighting individual oligomers. The traditional way to analyze this data is to take knowledge of the mass of the two monomers along with the polymerization reaction involved and assign each individual peak in the spectrum to a particular composition, typically several hundred peaks for a condensation-hydrolysis resin such as this. Although this may be the most thorough method to analyze the data it requires very high precision data and may not reveal significant trends in the data. Typically it is discovery of these trends and not accounting of each peak in the spectrum that is desired, especially in production quality control situations.

**Fig. 11:**
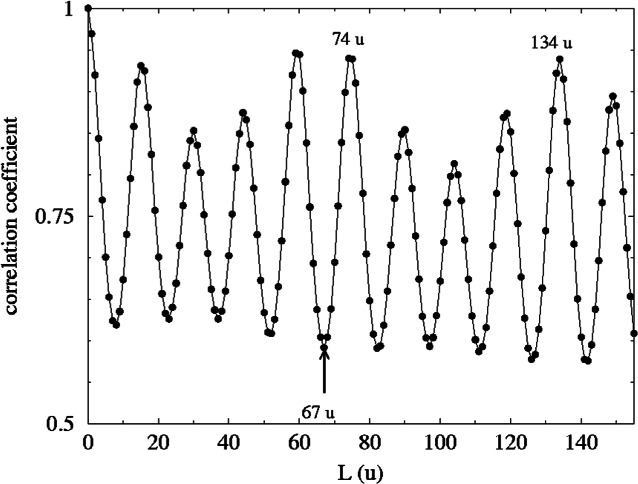
Mass autocorrelation of the data in Fig. 9. Labels indicate that the silsesquioxane repeat unit only appears as a dimer (134 u) and not as a monomer (67 u) while the siloxane repeat unit does appear as a monomer (74 u).

Figure 11 shows the autocorrelation of the data in Fig. 9. Peaks appear in the autocorrelation at each of the repeat distances of the main spectrum. There is a large peak at 74 u indicative of the dimethyl siloxane unit (D). That is to say that there frequently occurs pairs of oligomer separated in mass by 74 u, i.e., that the higher mass oligomer has grown by one D unit. Interestingly there is no peak at 67 u, which is the mass of the methyl silsesquioxane unit (T). However, there is a peak at 134 u that is twice the mass the silsesquioxane unit (2T). This immediately indicates that each oligomer present has an even number of T units. (Actually, to show this you also need to observe that there are also peaks at 4T, 6T, 8T, etc., but not at 3T, 5T, 7T, etc.) Each of the other peaks in the autocorrelation can be shown to be linear differences of 2T and D units forming the general function *n*2T–*m*D. Table 1 shows some of these combinations at lower mass. Notice that for every combination there is a peak in the autocorrelation and there are no peaks in the autocorrelation that are not in Table 1. Since the interpolation was done at 1 u intervals there are uncertainties of about 1 u between the table and Fig. 11.

The next observation to be made is that there is no peak at 18 u in the autocorrelation. The hydrolysis-condensation reaction gives off water when two silanols combine to form a bridging oxygen. As discussed previously, in incompletely-condensed silsesquioxanes a strong autocorrelation peak is seen at 18 u indicative of oligomers with the same number of repeat units but different degrees of condensation. The lack of a peak at 18 u immediately indicates that either full *intra* molecular condensation of silanols has taken place, or no *intra* molecular condensation of silanols has taken place. Only an exact indexing of peaks in the mass spectrum (which can be quite time consuming) can answer this question, however, it seems unlikely that condensation can occur to polymerize the material (intermolecular condensation) with some concomitant intramolecular condensation also occurring [16]. Additionally, an even number of T units is a strong indication of complete condensation since an odd number of T units would always leave at least one silanol in the material leading to further condensation reactions.

## 6. Autocorrelation in Signal-to-Noise Determinations

Up to now the autocorrelation function has been applied over the whole range of the polymer spectrum to understand polymer structure. However, in addition to polymer structure it is also often used to calculate moments of the molecular mass distribution (see Appendix C). To do so it is important to find the low-intensity oligomer peaks at the extrema of the molecular mass distribution. To accomplish this consider the use of the autocorrelation over only a part of the polymer spectrum. (This is *not* the “partial” autocorrelation function often discussed in time series analysis.) This “windowed” autocorrelation, analogous to a windowed FFT, is useful to determine where the signal has returned to baseline, that is, where does the signal devolve into the noise. This is crucial in the calculation of molecular mass distributions (MMD) from mass spectral data as the low and high mass oligomers at the extremes of the distribution have a disproportionate effect on the calculation. Since the thrust of the NIST polymer mass spectrometry effort is to make such determinations of MMD from mass spectral data it is of primary importance to us.

**Fig. 12:**
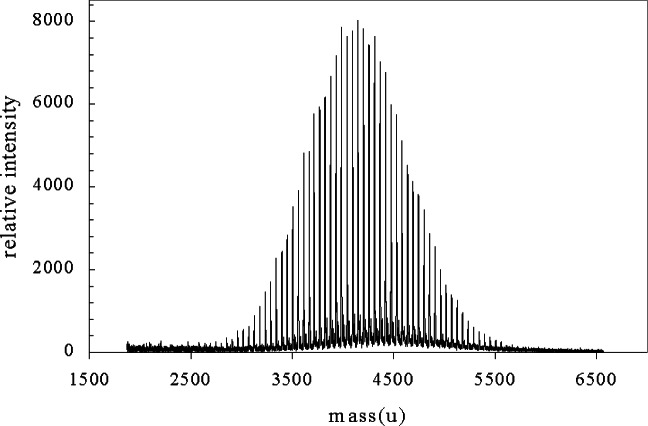
Matrix-assisted laser desorption/ionization time-of-flight mass spectrum from a polybutadiene of a molecular mass centered around 4100 u.

Figure 12 shows such a situation for polybutadiene (PBD, repeat unit: [–CH_2_–CH = CH–CH_2_–]). We propose to use the autocorrelation function to tell us more about where there is no signal in the noise. Let us say we use an integration window of a width 8 to 10 times the mass of the repeat unit and a maximum lag one half of the window length. Then we can move the integration window with increasing initial masses, *m*_i_, to higher and higher values. There will be a mass *m*_i_ where the correlation coefficient at the repeat unit mass will not rise above background. At this *m*_i_, we assume we have no signal while below it, we take it that we have signal. However, we must be careful about the baseline. If we have not taken the baseline off correctly, we will still see positive signal for the autocorrelation function not at the repeat unit. In fact, the baseline alone should be smooth signal between the repeat units with no peaks. Peaks should only appear at the repeat units. If they appear at other places at these high masses, we may suspect significant loss of an important signal (or perhaps a repeat unit present only at high mass).

**Fig. 13:**
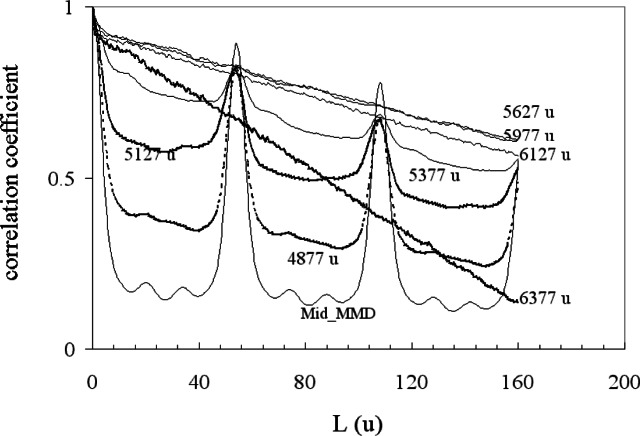
Mass autocorrelation of the data in Fig. 12 with windowing at the high mass edge of the mass spectra. The mass in the legend refers to the mass at the low edge of the window.

In Fig. 13 we apply our window choice on real polybutadiene data of Fig. 12 for about 10 repeat units (a range about 500 u wide) for lags out to nearly 3 repeat units starting each new window at 250 u increments with windows moving from 4877 u to the high molecular mass tail of the distribution. We notice a repeat unit in the window from the middle of the MMD at 54 u. This is the polybutadiene repeat unit mass. Additionally there are much weaker peaks at about 20 u and 34 u that are due to fragments along the chain backbone. For windows above *m*_i_ of 5377 u, we see no repeat unit signal at all. We then take our cut off of signal at 5627 u, the start of the next window. One might expect the autocorrelation function of a baseline of pure noise to be zero but it is not. If the noisy baseline were offset by a constant, the autocorrelation function would be unity. The linear autocorrelation function indicates an essentially constant baseline in time (see Appendix B).

**Fig. 14:**
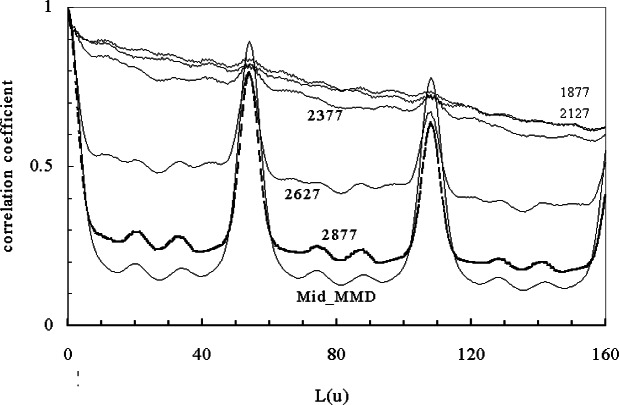
Autocorrelation function windowing at the low mass edge of the mass spectra. The mass in the legend refers to the mass at the low edge of the window.

**Fig. 15:**
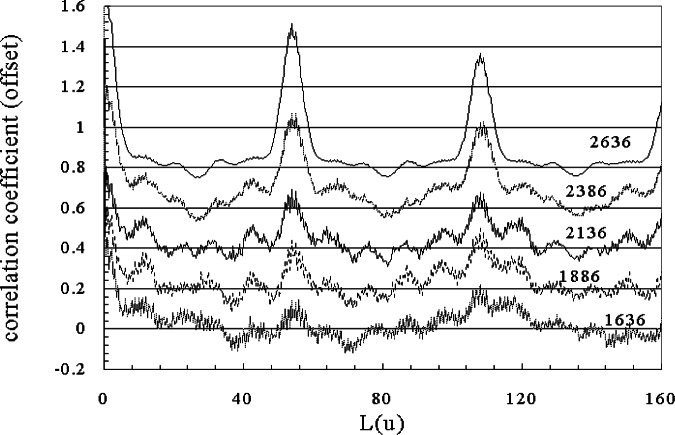
Autocorrelation function windowing at the low mass edge of the mass spectra after redrawing of the baseline. Compare to Fig. 12 where little signal is noticeable between the 1877 u window and the 2377 u window. Even the lowest window starting at 1636 u going to 2136 u shows mass signal at the repeat unit. It is mixed in with other repeats not identified yet. The mass in the legend refers to the mass at the low edge of the window.

In Fig. 14 we apply the same window width on the same data with windows moving toward the low tail of the distribution. Again, we notice a repeat unit in the window taken from the middle of the MMD at 54 u as well as much weaker peaks at about 20 u and 34 u. For windows with masses above 2127 u, we see only a repeat unit signal. Below this we may see some signal. Clearly here, the baseline signal is causing difficulty so we have redrawn the baseline for this data and the autocorrelation functions for windows starting at mass 1636 u are shown in Fig. 15. Once we draw a more correct baseline (i.e., through the noise in the spectra), the balancing of noise and the signal become clearer. For the peaks at mass 54 u on window 1636 u to 2386 u there are clearly peaks and some new peaks appear, apparently the appearance of another repeating species perhaps matrix clusters or silver cation clusters [17]. In this particular polymer, the average mass of silver (107.88 u), introduced as a cationizing agent, is about the same as two polybutadiene repeat units, confusing the issue somewhat.

## 7. Conclusion

We have shown that the autocorrelation function applied to the mass spectra of synthetic polymers allows one to more easily gain insight into the polymer single-chain structure. This offers a tool for looking at homopolymers with architectural changes like the silsesquioxanes and at the structure of complex polymers like the siloxane-silsesquioxane copolymer presented. Finally, we have shown how the windowed autocorrelation function can be used to separate signal from noise.

## Figures and Tables

**Fig. 16:**
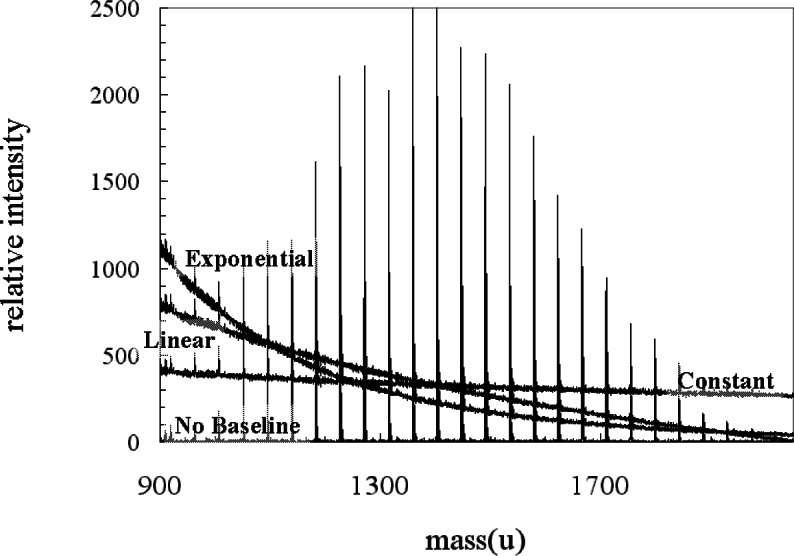
Transformation of mass axis with from equally-spaced points in time to equally-spaced points in mass with no added baseline in time, with a constant added baseline in time, with a linear added baseline in time, and with an exponential added baseline in time.

**Fig. 17:**
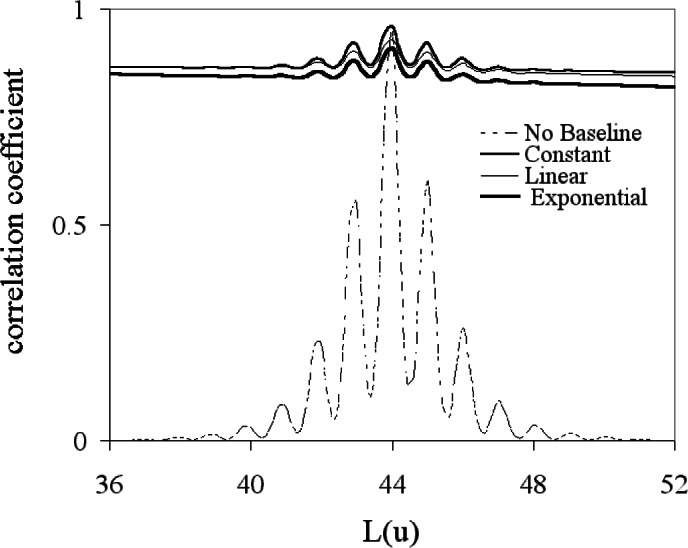
Autocorrelation function from equally-spaced points in mass space with no added baseline in time, with a constant added baseline in time, with a linear added baseline in time, and with an exponential added baseline in time.

**Fig. 18:**
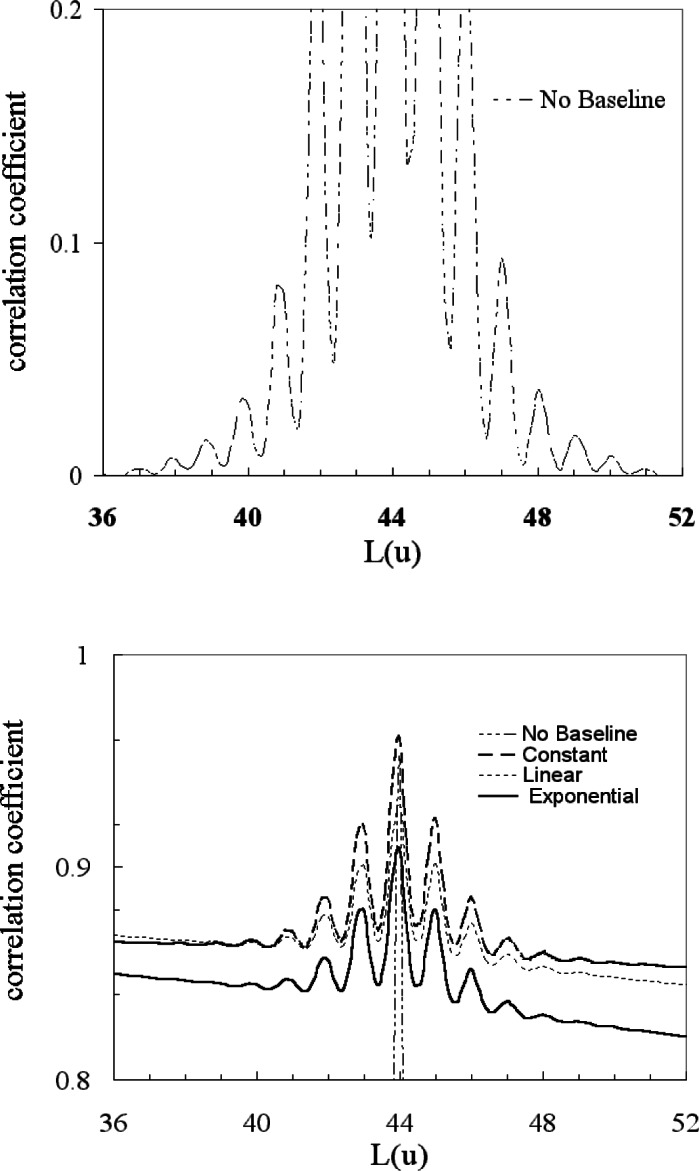
Expanded view of the mass autocorrelation shown in Fig. 17. Notice that 8 isotope peaks can be seen. This is derived from the observation that there are 17 peaks total which is equal to 2 · 8 + 1.

**Fig. 19:**
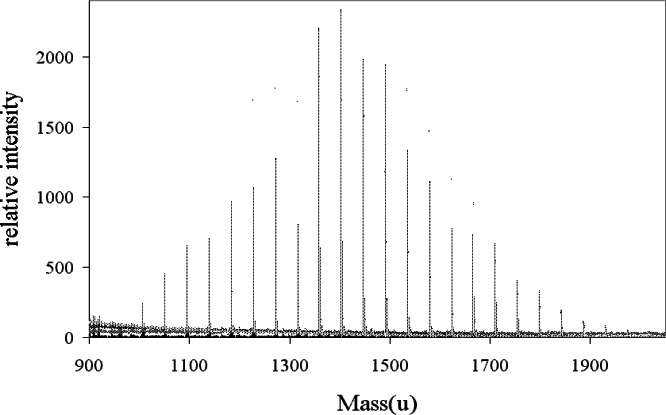
Redrawn baseline for the data in Fig. 12. Even small changes in baseline that have a minimal impact of the appearance of the spectrum can have a significant impact on calculating the molecular mass distribution (see Table 3).

**Table 1:** Identification of peaks in the mass autocorrelation of the copolymer resin using |*n*2T−*m*D|

*m*	*n*				
	0	1	2	3	4
0	0 u	134 u	268 u	402 u	536 u
1	74 u	60 u	194 u	328 u	463 u
2	148 u	14 u	120 u	254 u	389 u
3	222 u	88 u	46 u	180 u	315 u
4	296 u	162 u	28 u	106 u	240 u

**Table 2:** Computed MMD moments for various baselines with *A* = 100 in Eqs. (6), (7), and (8)

	*M*_n_ (u)	*M*_w_ (u)	*M*_z_ (u)
Without baseline	1424	1447	1468
Constant baseline	1420	1504	1580
Linear baseline	1229	1290	1353
Exponential baseline	1174	1239	1313

**Table 3:** Computed MMD moments for various baselines with *A* = 10 in Eqs. (6), (7), and (8)

	*M*_n_ (u)	*M*_w_ (u)	*M*_z_ (u)
Without baseline	1424	1447	1468
Constant baseline	1422	1486	1546
Linear baseline	1289	1343	1395
Exponential baseline	1254	1315	1375

## Footnotes

1 Certain commercial equipment, instruments, or materials are identified in this paper to foster understanding. Such identification does not imply recommendation or endorsement by the National Institute of Standards and Technology, nor does it imply that the materials or equipment identified are necessarily the best available for the purpose.

## 8. Appendix A: Transforming From Time to Mass When Using Time-of-Flight Mass Separation

To obtain correctly *S*(*m_i_*) from *s*(*t_i_*), the mass-based signal from the time-based signal, for the purpose of autocorrelation both a multiplicative factor equivalent to the Jacobean transform and subsequent interpolation are needed. Recall that while the points in time are evenly spaced conversion to mass places the data on a square-root point spacing. The easiest and most convenient method is to take the original data in time space and convert it to mass space with no signal conversion using a normal calibration program (this is what most commercial data programs output). This data is then interpolated onto equal mass intervals using a simple function with nothing being done to the signal intensity axis. The autocorrelation function is then taken on this data. This is simple but not rigorously correct; however, in our experience it gives a good representation and we regularly use it as a first approximation. Furthermore, this method will work with or without a subtracted baseline off although as seen below there are some small effects of the baseline of broadening of peaks.

A second more accurate method is to multiply these interpolated signal points which are equally spaced on the mass axis by dm/dt from the calibration curve [18]. Although this gives a correct signal it may give an incorrect representation of the noise (it will multiply the noise error by the factor dm/dt and will magnify any early time baseline by the same factor). This method is only rigorously correct if a baseline is pulled off the data; however, it will determine the autocorrelation function peaks correctly.

A third method is equivalent to the second in that it represents the data correctly on the mass scale. The protocol is as follows: go to the highest mass in the spectrum and determine the δ*m* between it and the next closest mass, essentially

$${\left(\delta m\right)}_{\text{max mass}}={\left(dm/dt\right)}_{\text{max mass}}\cdot \delta t$$ (4)

where δ*t* is the time interval of the digitizer and (d*m*/d*t*)_max mass_ is the calibrating derivative evaluated at the maximum mass in the spectrum. Then partial integrals are taken on the data over some interval larger than (δ*m*)_max mass_. This is now viewed as the new data. Only the issue of interpolation of the time data to obtain the partial integrals is a problem with this technique.

The reader should note there is a loss of information as one goes from equal-time-interval data to equal-mass-interval data, due to the nature of the *m α t*^2^ function. The integration or interpolation must be done with δ*m* larger than (δ*m*)_max mass_, the mass difference for one time unit at the highest mass considered. Otherwise, we are interpolating into a region where there is no signal.

## 9. Appendix B. Effect of Some Simple Model Baselines on the Mass Autocorrelation Function

Assume that the signal for any synthetic polymer is given by a sum of the signal from the baseline and a signal from the molecular mass distribution (MMD) of the polymer itself. We assume here as usual that the contribution from the baseline is additive to the true signal, then the total signal as received in time space, *S*_T_(*t_i_*), is:

$$S_{T}(t_{i})=S_{b}(t_{i})+S_{p}(t_{i})$$ (5)

where *S*_b_(*t_i_*) is the signal from the baseline and *S*_p_(*t_i_*) is the signal from the polymer. The baselines are added to the data in time space. As we shall see there is an effect of converting from time to mass space even for the baseline.

We take for the signal of the polymer the simple polyethylene oxide (PEO) spectrum given in Fig. 1 but now in time space. We have carefully pulled the baseline off before using it. This can be seen from Fig. 16 where on the signal conversion to mass space from time space using the partial integration method with no baseline pull off we see the signal in mass space is also at zero.

Here we present three models for a baseline. These signals are given in time space and are added to the PEO signal in time space. Since the baseline naturally occurs in time space, it seems most appropriate to offer a baseline in time space and transform it to mass space.

The most naive baseline model is a constant offset in time space,

$$S_{b}(t_{i})=A$$ (6)

where *A* is a constant independent of time. For the calculation given we chose *A* = 100. Notice our maximum signal in Fig. 1 for the PEO is 800 so this is a substantial baseline offset. It is our experience that this is not unusual.

Our second chose is a baseline linearly decreasing in time *t_i_*

$$S_{b}(t_{i})=2\cdot A\cdot (N_{\text{points}-i})/N_{\text{points}}$$ (7)

where *N*_points_ is the number of points in time space, *i* is the index of the time *t_i_* and 2 was chosen to keep the integral of this baseline signal identical to that of the constant baseline model above.

The third model is a decreasing exponential baseline. This is the one most commonly experienced in MALDI TOF mass spectrometry and is thought to be a result of matrix ions that are not energy focused. For this we chose:

$$S_{b}(t_{i})=3\cdot \exp(-3\cdot i/N_{\text{points}})$$ (8)

where *N*_points_ is the number of points in time space, *i* is the index of the time *t_i_* and 3 was chosen to keep the integral of the baseline signal identical to that of the constant offset model (and is set by the choice of the decay exponential as 3). Choice of the decay constant as 2 or smaller gave results close to that of the linear, as would be expected.

In Fig. 16 we show the signal for the various baselines converted to mass space. These show an effect of the baseline on converting from time to mass space. As would be expected partial integration over a varying width of integral space as we go from high mass to low mass over a constant window will lead us to not a constant baseline in mass space for a constant time baseline but one essentially linear in time. This is easily seen from the figure: the linear-in-time baseline leads to an apparent quadratic baseline in mass. For the exponential baseline it is not clear what it should lead to upon conversion to but most likely a modified exponential.

The autocorrelation function for these various choices of baseline is shown in Fig. 17. What is clear is pulling off the baseline gives one a much better representation to study the autocorrelation function. In Fig. 18 we expand the low region of the autocorrelation without baseline and show we can see up to at least eight masses of the isotope distribution. For the autocorrelation with baseline when we expand that region we can see at best six of these isotope regions. Thus even in the expanded region the addition of a non-zero baseline blurs the signal even when all significant figures are kept.

Finally we look at what the baseline does to the moments of the MMD. In Table 2 we show the change in the MMD moments as we change baseline. We notice that this is a little unfair since not unexpectedly the total area in the spectra with baselines is almost five times the area in the spectra without. Still the effect is striking and the lesson taken away is very important: leaving in a constant baseline in time affects the *M*_w_ and *M*_z_ moments significantly since these are dominated by the higher masses in the spectrum. Quadratic and cubic baseline functions contribute to increasing the higher moments by 3 % to 7 %. By leaving in the linear and exponential in time baselines, the quadratic and cubic mass contributions are overwhelmed by the excess contribution of the baseline at lower molecular masses.

Even if we choose a much smaller baseline the effect is significant. For example choose a baseline offset of *A* = 10 instead of the *A* = 100 signal units chosen in the discussion above. Figure 19 shows that the spectra all look quite similar. But the moments are affected significantly as seen in Table 3. For the simple exponential decay of the baseline in time, we see effects on *M*_n_ of 12 %.

## 10. Appendix C: Mass Autocorrelation Function as Estimated From the Mass-Average MMD Rather Than the Number-Average MMD

The normal autocorrelation function discussed in earlier sections has been an autocorrelation function on the number-average MMD as obtained from simply the spectra corrected as discussed above. This is the number-average MMD because under ideal circumstances we assume that the integrated area under any peak is proportional to the number of n-mers at that repeat unit. Another distribution commonly used in polymer science is the mass-weighted-average molecular mass distribution. This is the fraction of the mass at a given molecular mass. This is the MMD, which is usually obtained by size-exclusion chromatography using an ultraviolet or refractive index detector.

The autocorrelation function defined from this mass MMD is

$$G(l)=\Sigma \{[f(m_{i})\times m_{i}]\times [f(m_{i}+lm)\times (m_{i}+lm)]\}/\Sigma \{[f(m_{i})\times m_{i}]\times [f(m_{i})\times m_{i}]\}$$ (9)

where *f_i_* be the correctly normalized fraction for the number-average MMD. We have used this in a windowing program described in Sec. 6 looking specifically at the high mass region to see if weighting the distribution by the mass would make one choose a different window to consider in which the repeating mass started. Using this distribution we found no change from Fig. 13.

Finally often in studies of the autocorrelation function the mean value of the spectrum is pulled off the value at a given *i*th n-mer. Although we might have done this in our exploration of the baseline studies, there is no intrinsic physical or chemical meaning to the mean value of the number fractional spectrum. For a correctly normalized mass-weighted-average MMD is the mean value of mass, the *M*_n_ of the polymer and in considering the autocorrelation function in Eq. (9) it may be worth considering looking at it with *M*_n_ pulled off.
